# Relation between TLR4/NF-κB signaling pathway activation by 27-hydroxycholesterol and 4-hydroxynonenal, and atherosclerotic plaque instability

**DOI:** 10.1111/acel.12322

**Published:** 2015-03-10

**Authors:** Simona Gargiulo, Paola Gamba, Gabriella Testa, Daniela Rossin, Fiorella Biasi, Giuseppe Poli, Gabriella Leonarduzzi

**Affiliations:** Department of Clinical and Biological Sciences, School of Medicine, University of Turin, OrbassanoTurin, Italy

**Keywords:** atherosclerosis, 27-hydroxycholesterol, 4-hydroxynonenal, TLR4, cytokines, MMP-9

## Abstract

It is now thought that atherosclerosis, although due to increased plasma lipids, is mainly the consequence of a complicated inflammatory process, with immune responses at the different stages of plaque development. Increasing evidence points to a significant role of Toll-like receptor 4 (TLR4), a key player in innate immunity, in the pathogenesis of atherosclerosis. This study aimed to determine the effects on TLR4 activation of two reactive oxidized lipids carried by oxidized low-density lipoproteins, the oxysterol 27-hydroxycholesterol (27-OH) and the aldehyde 4-hydroxynonenal (HNE), both of which accumulate in atherosclerotic plaques and play a key role in the pathogenesis of atherosclerosis. Secondarily, it examined their potential involvement in mediating inflammation and extracellular matrix degradation, the hallmarks of high-risk atherosclerotic unstable plaques. In human promonocytic U937 cells, both 27-OH and HNE were found to enhance cell release of IL-8, IL-1β, and TNF-α and to upregulate matrix metalloproteinase-9 (MMP-9) via TLR4/NF-κB-dependent pathway; these actions may sustain the inflammatory response and matrix degradation that lead to atherosclerotic plaque instability and to their rupture. Using specific antibodies, it was also demonstrated that these inflammatory cytokines increase MMP-9 upregulation, thus enhancing the release of this matrix-degrading enzyme by macrophage cells and contributing to plaque instability. These innovative results suggest that, by accumulating in atherosclerotic plaques, the two oxidized lipids may contribute to plaque instability and rupture. They appear to do so by sustaining the release of inflammatory molecules and MMP-9 by inflammatory and immune cells, for example, macrophages, through activation of TLR4 and its NF-κB downstream signaling.

## Introduction

Atherosclerosis is a multifactorial disease characterized by the accumulation of lipids in the vascular wall. Although increased plasma lipids are central to the development of atherosclerosis, this disease is now mainly thought to be the consequence of a complex inflammatory process, involving immune responses at the initiation and progression of the atherosclerotic lesion, as well as at the time of plaque instability (Hansson, 2001; Steinberg, 2002). Several studies have suggested that atherogenesis is dependent on the innate immune response and that monocytes/macrophages, as well as T lymphocytes, all of which are cellular components of innate immunity, play predominant roles in atherosclerosis. Activation of these immune cells leads to a cascade of pro-inflammatory molecules release, which induces an inflammatory state in the vessel wall, with subsequent activation of the other arterial wall cells. Further, macrophages express receptors that recognize molecular patterns commonly found on pathogens (PAMPs: pathogen-associated molecular patterns) but foreign to mammalian organisms and are important in innate immunity. In this context, the type I transmembrane receptors known as Toll-like receptors (TLRs) are the innate immune receptors that have been most extensively studied and characterized. TLRs, however, are not only expressed on macrophages but also on the other cells commonly found in the arterial wall (Seneviratne *et al*., 2012).

A variety of exogenous and endogenous ligands can activate TLRs by binding the extracellular domain: This activation induces dimerization of the receptor and binding of either the adaptor protein myeloid differentiation factor 88 (MyD88) or the TIR domain-containing adaptor inducing interferon-β (TRIF)-dependent pathway, which process culminates in interferon-regulating factor 3 (IRF3) activation. Indeed, TLRs require either the MyD88-dependent (all TLRs except TLR3) or the TRIF-dependent (TLR4 and TLR3) pathway to initiate downstream signaling. This then leads to mitogen-activated protein kinases (MAPKs) and nuclear factor-κB (NF-κB) activation, with consequent release of a variety of inflammatory molecules, thus augmenting the local inflammatory response and/or matrix breakdown. TLRs, thus, play a crucial role in the initiation of an innate immune response through activation of the inflammatory cells via NF-κB-dependent pathway and in the subsequent activation of the adaptive immune response (Jeong & Lee, 2011; Bijani *et al*., 2012).

There is increasing evidence to support the involvement of TLRs, mainly TLR2 and TLR4, in the initiation, progression, and instability of atherosclerotic lesions, leading to plaque rupture (Edfeldt *et al*., 2002; Vink *et al*., 2004; Schoneveld *et al*., 2008; Curtiss & Tobias, 2009; Seneviratne *et al*., 2012; Cole *et al*., 2013), as well as their involvement in other cardiovascular diseases (Bijani *et al*., 2012). However, their functional role in human atherosclerosis is still unclear.

TLR4 overexpression has been reported in both human and mouse atherosclerotic lesions, mainly in macrophages and endothelial cells (ECs) within the lesion, at different stages of atherogenesis (Xu *et al*., 2001; Edfeldt *et al*., 2002; Vink *et al*., 2002; Pasterkamp *et al*., 2004; Miller *et al*., 2009; den Dekker *et al*., 2010). Activation of TLR4 upregulates the expression of various cytokines, chemokines, and adhesion molecules involved in cell recruitment and proliferation, thus initiating the inflammatory response and promoting intimal foam cell accumulation. However, although the role of TLR4 is well established in the early phase and in the progression of atherosclerosis, as shown by studies in knockout mice (Björkbacka *et al*., 2004; Michelsen *et al*., 2004; Higashimori *et al*., 2011), its role in advanced and unstable atherosclerotic plaques has yet to be fully assessed, and few studies point to an association between TLR4 activation and plaque rupture (Methe *et al*., 2005; Geng *et al*., 2006; Ishikawa *et al*., 2008). TLR4 has been shown to be important in the process of expansive arterial remodeling and in matrix breakdown; the latter process involves cell migration and leads to higher expression levels of matrix metalloproteinases (MMPs), mainly MMP-2 and MMP-9, which are involved in extracellular matrix (ECM) degradation (Hollestelle *et al*., 2004).

Alongside the classic endogenous ligands, the endogenously produced TLR ligands most relevant to the context of atherosclerosis are the minimally modified low-density lipoproteins (mmLDLs) and the oxidized LDLs (oxLDLs), together with their active components; these include oxidized phospholipids, cholesterol oxidation products (oxysterols), and free and esterified aldehydes.

It has been demonstrated that both mmLDLs and oxLDLs activate murine and human monocytes and macrophages during inflammatory processes, in a mechanism involving TLR2 and TLR4, and that they increase the expression of these receptors so as to enhance and sustain pro-inflammatory molecule production (Xu *et al*., 2001; Chávez-Sánchez *et al*., 2010).

Among the oxidized lipids carried by oxLDLs, cholesterol oxidation products, including the most abundant circulating oxysterol 27-hydroxycholesterol (27-OH), and one of the more reactive end products of lipid peroxidation, namely 4-hydroxynonenal (HNE), play a key role in the pathogenesis of atherosclerosis by stimulating various signal transduction pathways, involved in the immune and inflammatory response as well as in oxidative stress. These are all important events that contribute to enhancing MMP production and activation, resulting in excessive matrix degradation and the rupture of vulnerable atherosclerotic plaques (Leonarduzzi *et al*., 2005; Poli *et al*., 2009; Gargiulo *et al*., 2011). Of note, among the various MMPs, MMP-9 has been consistently implicated in the pathophysiology of plaque rupture, and various inflammatory molecules, in particular interleukin-6 (IL-6), IL-8, IL-1β, and tumor necrosis factor-α (TNF-α), play crucial roles in the cross talk between vascular cells leading to upregulated MMP expression (Moreau *et al*., 1999; Lu *et al*., 2012; Zhong *et al*., 2014).

Within this framework, the objective of this study on human promonocytic U937 cells was to examine the effects of the oxysterol 27-OH and the unsaturated aldehyde HNE on TLR4 activation. These two oxidized lipids, which have important pathophysiological actions *in vivo*, are consistently involved in the pathogenesis of atherosclerosis due to their substantial accumulation in the atherosclerotic plaque where they act as potent inducers of inflammatory gene expression (Leonarduzzi *et al*., 2005; Poli *et al*., 2008, 2009; Sottero *et al*., 2009). Moreover, in our previous study, 27-OH was found to be the principal oxysterol responsible for the MMP-9 upregulation (Gargiulo *et al*., 2011). It also looked at the potential involvement of TLR4 in mediating inflammation and matrix degradation during the progression of atherosclerosis. Both oxidized lipids were found to enhance the release of IL-8, IL-1β, and TNF-α and to upregulate MMP-9, via TLR4/NF-κB-dependent pathway, thereby augmenting both inflammatory response and matrix degradation, which lead to atherosclerotic plaque instability and eventually rupture. Given that an inflammatory state can contribute to MMP production, it is of note that using specific antibodies, it was also demonstrated that these inflammatory cytokines act on MMP-9, upregulating its expression and synthesis, thus sustaining the release of this matrix-degrading enzyme by vascular cells, and contributing to plaque instability.

## Results

### Activation of TLR4 and NF-κB by 27-hydroxycholesterol and 4-hydroxynonenal in U937 cells

To investigate whether 27-OH and HNE affect the activation of TLR4, human promonocytic U937 cells were incubated with these two components of oxLDLs. The oxysterol 27-OH was used at 6 μm, a concentration mimicking the amount of this oxysterol found in atherosclerotic plaques (Leonarduzzi *et al*., 2007), while HNE was used at a concentration of 5 μm, which is below that found in inflamed and diseased tissues (Esterbauer *et al*., 1991), to avoid alteration and/or inhibition of various cellular responses (Poli *et al*., 2008). Moreover, at these concentrations, these compounds were not cytotoxic as demonstrated by MTT analysis and trypan blue exclusion test (data not shown).

**Fig 1:**
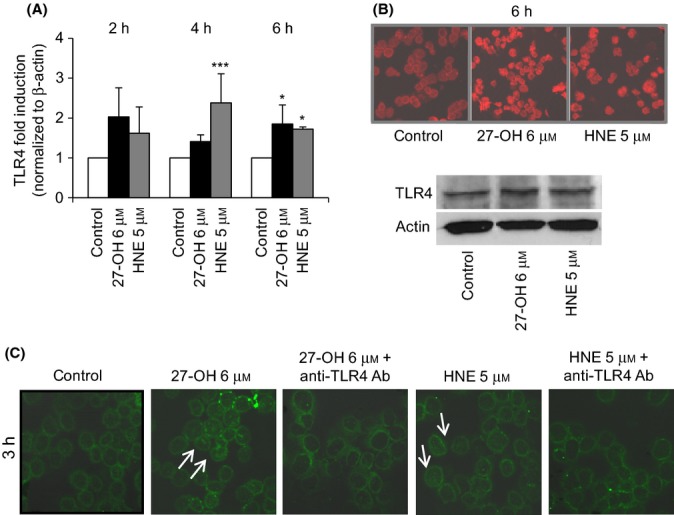
Activation of TLR4/NF-κB pathway by 27-OH and HNE in U937 cells. Cells were incubated with 6 μm 27-OH or 5 μm HNE. (A) TLR4 expression was quantified after incubation with 27-OH or HNE by real-time RT–PCR for up to 6 h. Data are means ± SD of four experiments and are expressed as fold induction vs. control (untreated cells). ***P < 0.001 and *P < 0.05 vs. control. (B) After cell treatment for 6 h, TLR4 protein levels were detected by confocal laser microscopy using a TRITC-conjugated secondary antibody (532-nm exciting laser band, 572-nm long-pass emission filter, and 40x/0.75 lens). The images are representative of three experiments. TLR4 protein levels were also analyzed by Western blotting after 6-h cell treatment. The blot is representative of two experiments. (C) After cell co-treatment with 0.2 μg mL^-1^ TLR4 antibody and with 27-OH or HNE for 3 h, cytoplasmic and nuclear localization of p65, a subunit of NF-κB, was visualized by confocal laser microscopy using a FITC-conjugated secondary antibody (488-nm exciting laser band and emission passing through a long-pass 505-550 filter, lens 40x/0.75). The images are representative of three experiments.

The effects of 27-OH and HNE on TLR4 expression were quantified by real-time RT–PCR. An approximately time-dependent increase of TLR4 mRNA levels was observed from 2 to 6 h of treatment, compared with untreated cells (control). Both oxidized lipids significantly induced TLR4 expression after 6-h cell treatment (Fig.1A). An increase of TLR4 protein levels was observed by immunofluorescence and by Western blotting analysis after 6-h cell incubation with either 27-OH or HNE (Fig.1B).

Because TLR4 is a key inducer of the NF-κB pathway, which is one of the principal inflammatory signals, the relationship between TLR4 and NF-κB signaling pathways was investigated. One of the key events in NF-κB activation involves phosphorylation of IκB followed by nuclear translocation of p65, a subunit of the NF-κB complex. Nuclear translocation of the p65 subunit was demonstrated by immunofluorescence analysis (using a confocal laser microscope) in U937 cells after 3-h treatment with either 27-OH or HNE. Translocation of p65 into the nucleus was evident, compared with control cells, in both cases, but was more pronounced after 27-OH cell incubation (Fig.1C). To verify the relationship between TLR4 and NF-κB signaling pathways, cells were incubated with 27-OH or HNE in the presence (co-treatment) of an anti-TLR4 antibody (0.2 μg mL^-1^). By blocking the TLR4 pathway, nuclear translocation of p65, as downstream signaling, was fully inhibited (Fig.1C).

### Upregulation of IL-8, IL-1β, and TNF-α by 27-OH and HNE

Among the inflammatory cytokines involved in the pathogenesis of atherosclerosis and associated with matrix degradation, the effect of 6 μm 27-OH and 5 μm HNE on IL-8, TNF-α, and IL-1β expression and protein levels were checked in U937 cells, by quantitative RT–PCR for up to 8-h treatment and by Bio-Plex® system after 24-h treatment, respectively.

**Fig 2:**
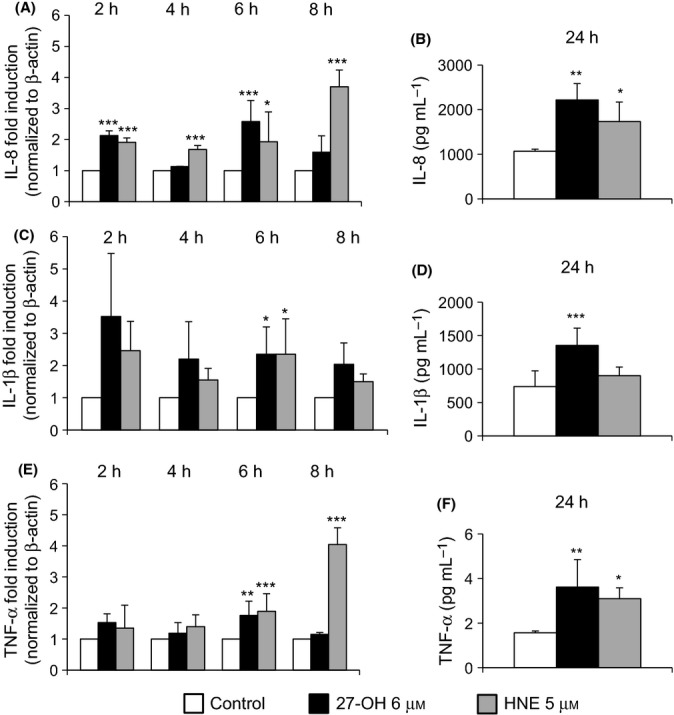
27-OH and HNE induce expression and synthesis of IL-8, IL-1β, and TNF-α. Expression of (A) IL-8, (C) IL-1β, and (E) TNF-α was quantified by real-time RT–PCR in U937 cells treated with 6 μm 27-OH or 5 μm HNE for up to 8 h. The histograms represent the mean values ± SD of four experiments, expressed as fold induction versus control (untreated cells). ***P < 0.001, **P < 0.01, and *P < 0.05 vs. control. Levels of (B) IL-8, (D) IL-1β, and (F) TNF-α protein were analyzed by the Bio-Plex® system. Cytokine concentrations (pg mL^-1^) were extrapolated from the standard curve. The histograms represent the mean values ± SD of three experiments. ***P < 0.001, **P < 0.01, and *P < 0.05 vs. control.

The expression of IL-8 was significantly increased by 27-OH after 2- and 6-h treatment, while HNE induced IL-8 expression in a significant manner at all the times examined (Fig.2A). The protein levels of this inflammatory cytokine were also found to be markedly higher in cells treated for 24 h with 27-OH or with HNE, compared with untreated cells (control) (Fig.2B). Regarding IL-1β, both 27-OH and HNE induced expression of the cytokine at all times investigated, but the increase was only significant after 6-h cell treatment (Fig.2C); data obtained with the Bio-Plex® system indicate that only 27-OH significantly increased IL-1β protein levels after 24-h treatment (Fig.2D). Lastly, expression of TNF-α was significantly induced by 27-OH at 6 h of incubation and by HNE in a time-dependent manner but with a significant increase at 6 and 8 h of incubation (Fig.2E). Protein levels of TNF-α were induced by both 27-OH and HNE (Fig.2F).

### Down-regulation of the inflammatory cytokines by inhibiting the TLR4/NF-κB signaling pathway

TLR4 plays an important role in the inflammatory signaling responses to various stimuli, leading to the transcription, via NF-κB-dependent pathway, of a variety of genes involved in inflammation.

To investigate the key association between activation of the TLR4/NF-κB pathway and cytokine upregulation, U937 cells were treated for 6 h (time point at which cytokine expression was consistently induced by both oxidized lipids) or for 24 h, with 6 μm 27-OH or with 5 μm HNE, in the presence or absence of an antibody directed against TLR4. To support the involvement of TLR4 in the inflammation process induced by 27-OH and HNE, the effect of TLR4 gene knockdown using a specific small interfering RNA (siRNA) was also investigated after 24 h of reverse transfection, followed by 6-h cell incubation with 27-OH or HNE.

**Fig 3:**
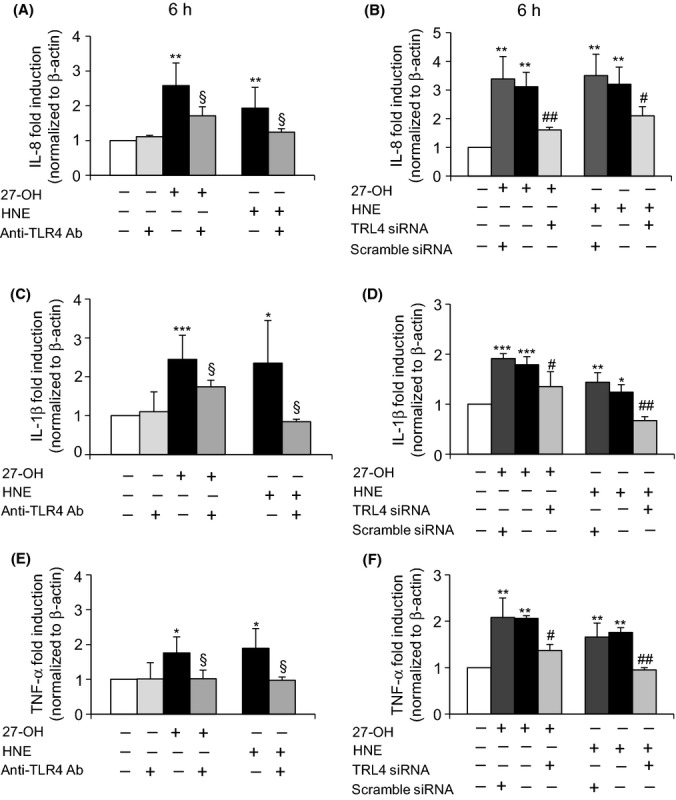
Blocking TLR4 activation negatively affects expression of IL-8, IL-1β, and TNF-α genes. U937 cells were either co-treated with 0.2 μg mL^-1^ TLR4 antibody or transfected with a specific TLR4 siRNA for 24 h and then incubated with 6 μm 27-OH or 5 μm HNE for 6 h. Scramble siRNA (negative control) corresponds to a siRNA with nonspecific sequence. Expression of (A,B) IL-8, (C,D) IL-1β, and (E,F) TNF-α was analyzed by quantitative RT–PCR. The histograms represent the means values ± SD of three experiments. ***P < 0.001, **P < 0.01, and *P < 0.05 vs. control; §*P* < 0.05 vs. 27-OH or HNE; ##*P* < 0.01 and #*P* < 0.05 vs. 27-OH or HNE.

**Fig 4:**
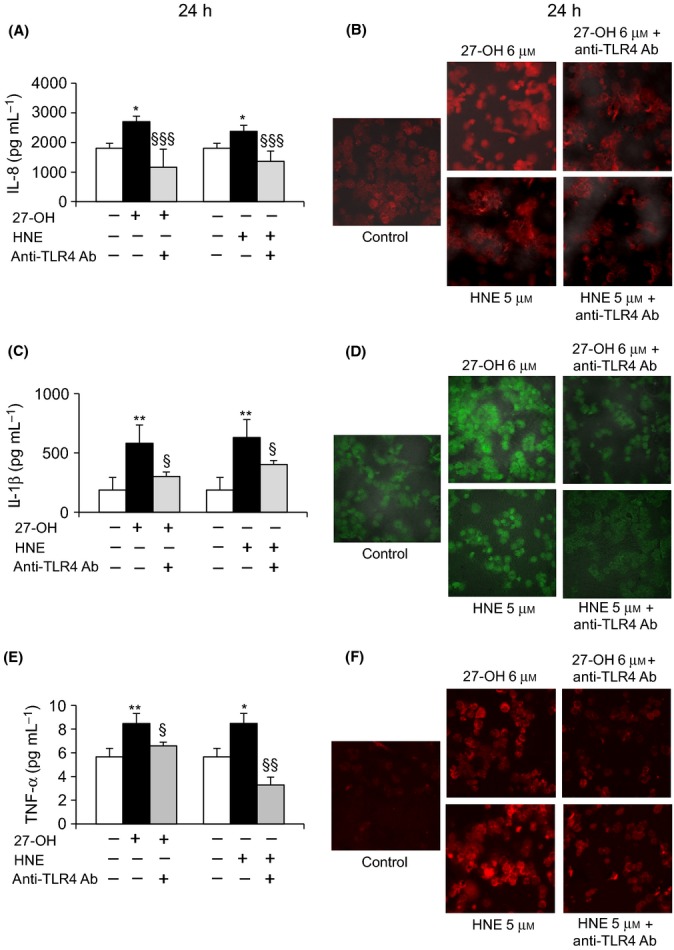
Effect of TLR4 inhibition on IL-8, IL-1β, and TNF-α protein levels. U937 cells were co-treated with 0.2 μg mL^-1^ TLR4 antibody and then incubated for 24 h with 6 μM 27-OH or 5 μM HNE. Protein levels of (A) IL-8, (C) IL-1β, and (E) TNF-α were analyzed using the ELISA method. Cytokine concentrations (pg mL^-1^) were extrapolated from the standard curve. The histograms represent the mean values ± SD of three experiments. **P < 0.01 and *P < 0.05 vs. control; §§§*P* < 0.001, §§*P* < 0.01, and §*P* < 0.05 vs. 27-OH or HNE. Immunopositive cells were detected by confocal laser microscopy: (B) IL-8 and (D) IL-1β using a TRITC-conjugated secondary antibody (532-nm exciting laser band, 572-nm long-pass emission filter, and 40x/0.75 lens); (F) TNF-α using a FITC-conjugated secondary antibody (488-nm exciting laser band and emission passing through a long-pass 505-550 filter, lens 40x/0.75). The images are representative of three experiments.

A significant decrease of expression of IL-8 (Fig.3A,B), IL-1β (Fig.3C,D), and TNF-α (Fig.3E,F) was observed in cells co-treated with an anti-TLR4 antibody (0.2 μg mL^-1^) as well as in cells transfected with a TLR4 siRNA, compared with cells treated with 27-OH or with HNE. Observation was carried out by real-time RT–PCR. Cell incubation with the anti-TLR4 antibody alone did not affect cytokine expression; cell transfection with a negative control (scramble siRNA) demonstrated a specific effect on gene expression of TLR4 siRNA. A marked and significant decrease of protein levels of IL-8 (Fig.4A,B), IL-1β (Fig.4C,D), and TNF-α (Fig.4E,F) was also observed in cells incubated with an anti-TLR4 antibody (0.2 μg mL^-1^). Observation was carried out by ELISA and immunofluorescence analysis using a confocal laser microscope.

**Fig 5:**
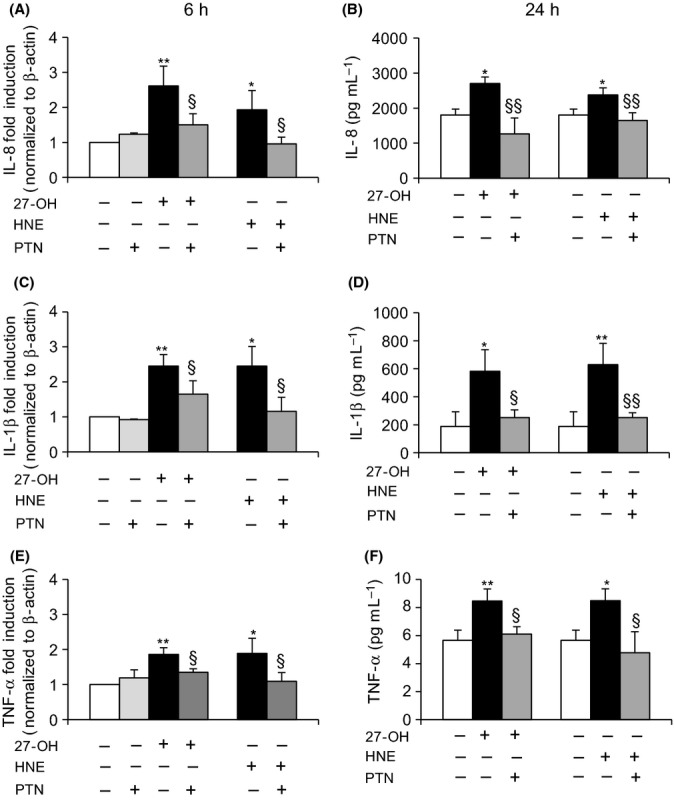
Effect of NF-κB inhibition on cytokine expression and protein levels. U937 cells were pretreated for 1 h with 1 μM parthenolide (PTN) and then with 6 μm 27-OH or 5 μm HNE. Expression of (A) IL-8, (C) IL-1β, and (E) TNF-α was analyzed by real-time RT–PCR after 6-h cell incubation with 27-OH or HNE. The histograms represent the mean values ± SD of five experiments. **P < 0.01 and *P < 0.05 vs. control; §*P* < 0.05 vs. 27-OH or HNE. Protein levels of (B) IL-8, (D) IL-1β, and (F) TNF-α were quantified by ELISA after 24-h cell incubation. Cytokine concentrations (pg mL^-1^) were extrapolated from the standard curve. The histograms represent the mean values ± SD of three experiments. **P < 0.01 and *P < 0.05 vs. control; §§*P* < 0.01 and §*P* < 0.05 vs. 27-OH or HNE.

**Fig 6:**
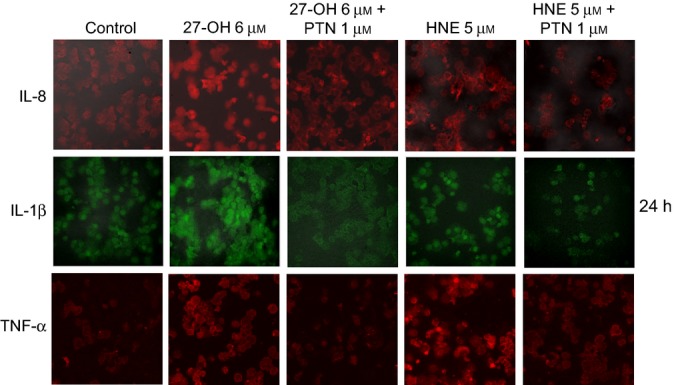
NF-κB inhibition decreases cytokine levels. U937 cells were pretreated for 1 h with 1 μm parthenolide (PTN) and then incubated with 6 μm 27-OH or 5 μm HNE for 24 h. Immunopositive cells were detected by confocal laser microscopy: IL-8 and IL-1β using a TRITC-conjugated secondary antibody (532-nm exciting laser band, 572-nm long-pass emission filter, and 40x/0.75 lens); TNF-α using a FITC-conjugated secondary antibody (488-nm exciting laser band and emission passing through a long-pass 505-550 filter, lens 40x/0.75). The images are representative of three experiments.

To test experimentally the involvement of the transcription factor NF-κB in the modulation of these inflammatory cytokines, other cells were pretreated (1 h) with 1 μm parthenolide (PTN), a specific inhibitor of NF-κB. Blocking NF-κB nuclear translocation with parthenolide caused a significant decrease in IL-8, IL-1β, and TNF-α expression, in cells treated for 6 h with either 27-OH or HNE (Fig.5A,C,E). Cell incubation with PTN alone did not affect cytokine expression. An evident decrease of protein levels of these inflammatory molecules was also detected by ELISA (Fig.5B,D,F) and confocal microscopy (Fig.6) after cell pretreatment with PTN followed by cell incubation with 27-OH or HNE for 24 h.

### Upregulation of MMP-9 by 27-OH and HNE in U937 cells via TLR4/NF-κB-dependent pathway

**Fig 7:**
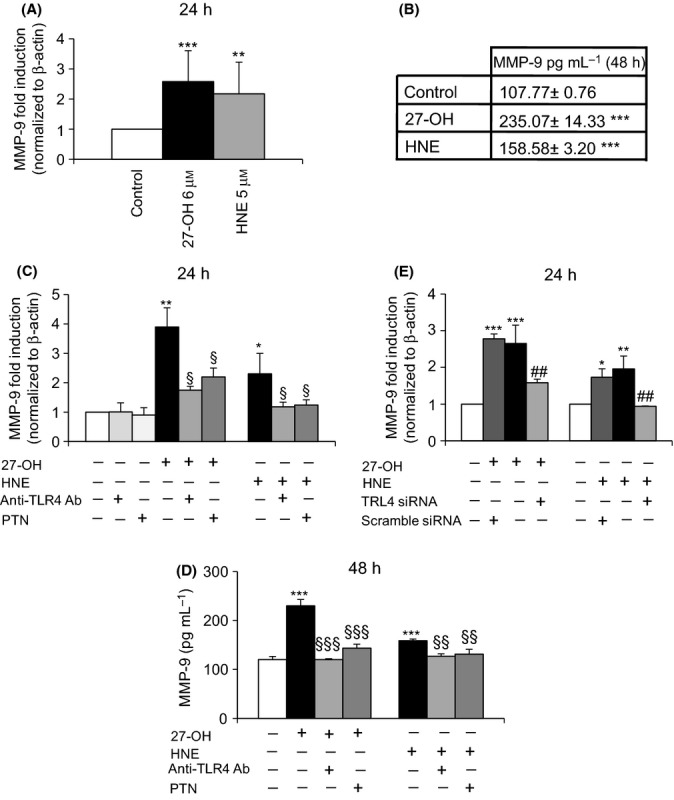
27-OH and HNE induce MMP-9 through the TLR4/NK-κB signaling pathway. (A) MMP-9 gene expression was evaluated by real-time RT–PCR after 24-h cell incubation with 6 μm 27-OH or 5 μm HNE. The histograms represent the mean values ± SD of three experiments. ***P < 0.001 and **P < 0.01 vs control. (B) Protein levels of MMP-9 were measured by ELISA after 48-h cell incubation. Data are means ± SD of four experiments; protein concentrations (pg mL^-1^) were extrapolated from the standard curve. ***P < 0.001 vs. control. (C) MMP-9 expression was analyzed by real time RT–PCR in U937 cells co-treated with 0.2 μg mL^-1^ anti-TLR4 antibody or pretreated with 1 μM parthenolide (PTN) and then incubated with 6 μm 27-OH or 5 μm HNE for 24 h. The histograms represent the mean values ± SD of three experiments. **P < 0.01 and *P < 0.05 vs. control; §*P* < 0.05 vs. 27-OH or HNE. (D) MMP-9 protein levels were measured by ELISA after 48-h cell incubation with 27-OH or HNE with or without co-treatment with an anti-TLR4 antibody or pretreatment with PTN. Data are means ± SD of three experiments. ***P < 0.001 vs. control; §§§*P* < 0.001 and §§*P* < 0.05 vs. 27-OH or HNE. (E) U937 cells were transfected for 24 h with a specific TLR4 siRNA, and MMP-9 expression was evaluated after 24 h of incubation with 27-OH or HNE by real-time RT–PCR. Scramble siRNA (negative control) corresponds to a siRNA with nonspecific sequence. Data are expressed as mean values ± SD of three experiments. ***P < 0.001, **P < 0.01, and *P < 0.05 vs. control; ##*P* < 0.01 vs. 27-OH or HNE.

The effects of 27-OH (6 μm) and HNE (5 μm) on MMP-9 expression and synthesis were checked by quantitative RT–PCR after 24-h treatment and by ELISA after 48-h treatment, respectively. Of note, the ELISA kit employed recognizes both inactive and active forms of MMP-9. In human promonocytic U937 cells, MMP-9 expression (Fig.7A) and protein levels (Fig.7B) were consistently induced both by the oxysterol and by the lipid aldehyde, compared with untreated cells (control).

As supported by other studies, MMP-9 release can be regulated via TLR4/NF-κB-dependent pathway (Ikeda & Funaba, 2003; Gebbia *et al*.,^2004^; Li *et al*., 2012; Paolillo *et al*., 2012).

The involvement of TLR4 in MMP-9 upregulation induced by 27-OH and HNE was investigated by blocking the receptor with a specific anti-TLR4 antibody and by TLR4 knockdown using a specific siRNA. Co-treatment of U937 cells with an anti-TLR4 antibody significantly decreased both mRNA (Fig.7C) and protein (Fig.7D) levels of MMP-9, as shown by quantitative RT–PCR and ELISA, respectively. To clarify involvement of the TLR4/NF-κB signaling pathways in the MMP-9 upregulation, other cells were also pretreated with 1 μm parthenolide (PTN). Pretreatment of U937 cells with this specific inhibitor of NF-κB nuclear translocation clearly and significantly decreased both expression (Fig.7C) and protein levels (Fig.7D) of MMP-9, induced by 27-OH and HNE. Cell incubation with the anti-TLR4 antibody or PTN alone did not affect the expression of MMP-9, compared with untreated cells. To support the involvement of TLR4 in MMP-9 expression, cells were transfected for 24 h with a specific TLR4 siRNA: A significant decrease of MMP-9 expression was found in U937 cells treated with both 27-OH and HNE for 24 h following TLR4 gene knockdown (Fig.7E). Cell transfection with a negative control (scramble siRNA) demonstrated a specific effect on gene expression of MMP-9 siRNA.

These data support the potential role of TLR4/NF-κB-dependent pathway in MMP-9 upregulation and subsequently in arterial remodeling and plaque instability.

### Involvement of inflammatory cytokines on MMP-9 upregulation

Besides the potential direct upregulation of MMP-9 through the TLR4/NF-κB-dependent pathway, as suggested by the above results, transcription of the MMP-9 gene might also be stimulated by inflammatory cytokine release following NF-κB activation, thus contributing to atherosclerotic plaque instability and rupture.

**Fig 8:**
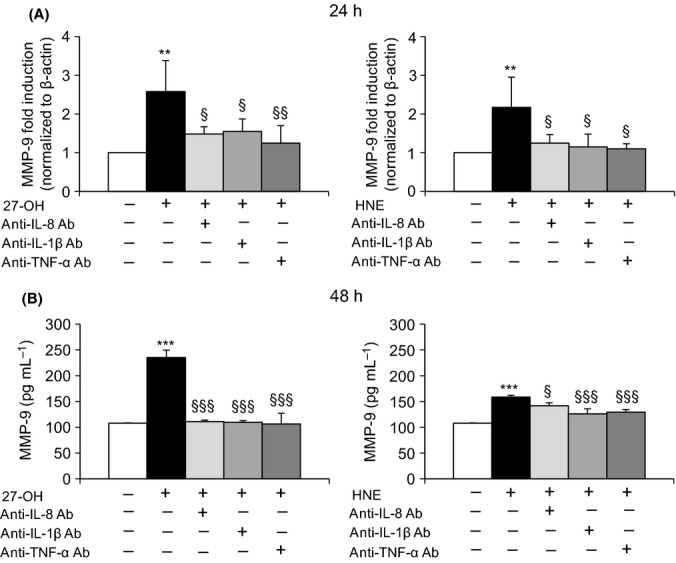
Effect of inhibiting cytokine release on MMP-9 levels. U937 cells were treated with 6 μm 27-OH or 5 μm HNE in the presence or the absence of 0.04 μg mL^-1^ of IL-8, IL-1β, or TNF-α primary antibodies. (A) MMP-9 expression was measured by real-time RT–PCR at 24 h. The histograms represent the mean values ± SD of three experiments and are expressed as fold induction versus control (untreated cells). **P < 0.01 vs. control; §§*P* < 0.01 and §*P* < 0.05 vs. 27-OH or HNE. (B) MMP-9 protein levels were measured by ELISA after 48-h cell incubation. Data are means ± SD of three experiments; protein concentrations (pg mL^-1^) were extrapolated from the standard curve. ***P < 0.001 vs. control; §§§*P* < 0.001 and §*P* < 0.05 vs. 27-OH or HNE.

To investigate this hypothesis, the action of IL-8, IL-1β, and TNF-α, which are released following cell treatment with 6 μm 27-OH and 5 μm HNE, was blocked with specific antibodies; both MMP-9 mRNA (Fig.8A) and protein (Fig.8B) levels were consistently decreased, confirming the importance of these cytokines in regulating the protease levels induced by 27-OH and HNE.

## Discussion

In the present study, we first demonstrated the effects of the oxysterol 27-OH and the unsaturated aldehyde HNE on TLR4 activation in human promonocytic U937 cells; both oxidized lipids accumulate in atherosclerotic plaques and play a key role in the pathogenesis of atherosclerosis. We also demonstrated the potential involvement of the TLR4/NF-κB signaling pathway in mediating inflammation and matrix degradation, thus contributing to atherosclerotic plaque instability and eventually rupture. 27-OH and HNE were indeed found to enhance the release of IL-8, IL-1β, and TNF-α and to upregulate MMP-9, via TLR4/NF-κB-dependent pathway. These data support the potential role of TLR4 and its NF-κB downstream signaling in inflammatory cytokines and MMP-9 upregulation, and subsequently in plaque instability.

In this connection, an increasing body of evidence points to an important role of TLRs in the pathogenesis of atherosclerosis. Arterial wall cells (mainly ECs, macrophages, and smooth muscle cells) constitutively express TLRs, whereas in the same cells, the expression of TLRs is upregulated in the presence of hyperlipidemia (Edfeldt *et al*., 2002; Schoneveld *et al*., 2008; Seneviratne *et al*., 2012). Among the key players in innate immunity and inflammatory signaling responses to exogenous and endogenous stimuli, TLR4 appears potentially involved in this multifactorial disease.

TLR4 over-expression has been described in human and mouse atherosclerotic lesions at different stages of atherogenesis in cells commonly found in the arterial wall, but especially in macrophages and ECs (Xu *et al*., 2001; Edfeldt *et al*., 2002; Vink *et al*., 2002; Pasterkamp *et al*., 2004; Miller *et al*., 2009; den Dekker *et al*., 2010). Supporting evidence for the role of TLRs in plaque progression and instability was found in a study on double knockout mice for apolipoprotein E (ApoE) and MyD88. After being fed a cholesterol-rich diet, the mice showed a reduction in aortic atherosclerosis of almost 55%: Lesion size, number of macrophages/foam cells, and lipid content in aortic plaques, as well as circulating inflammatory molecules, were all markedly reduced compared with *ApoE*^*−/−*^ and *MyD88*^*+/+*^ mice. Thus, in *ApoE*^*−/−*^ and *MyD88*^*−/−*^ mice, not only was the atherosclerotic lesion reduced, but the plaque phenotype was also altered. Of note, the major effect of MyD88 deletion on activated macrophage was at the level of pro-inflammatory molecule expression (Björkbacka *et al*., 2004; Michelsen *et al*., 2004; Higashimori *et al*., 2011). In another study, double knockout mice for ApoE and TLR4 (*ApoE*^*−/−*^*/TLR4*^*−/−*^) also showed markedly reduced atherosclerosis and altered plaque phenotype, despite persistent hypercholesterolemia (Michelsen *et al*., 2004). Collectively, these results indicate that inactivation of TLR4/MyD88-dependent signaling strongly inhibits development of atherosclerosis and promotes a more stable plaque phenotype, mainly by reducing inflammatory molecule release as a consequence of NF-κB inactivation.

TLR gene polymorphisms have also been studied in connection with atherosclerotic vascular disease. The Asp299Gly polymorphism in TLR4 has been associated with a lower risk of carotid atherosclerosis, and a reduction in intima media thickness in the common carotid artery (Kiechl *et al*., 2002; Hernesniemi *et al*., 2008), and has also been linked to decreased acute coronary events, and greater efficacy of statin therapy (Ameziane *et al*., 2003; Boekholdt *et al*., 2003). However, conflicting reports have also been published, showing that the presence or the absence of the Asp299Gly polymorphism is not associated with predisposition to, or progression of, atherosclerotic lesions in patients with hypercholesterolemia (Netea *et al*., 2004).

TLR4 has also been shown to be important in the process of expansive arterial remodeling and in ECM degradation leading to higher expression levels of MMPs, mainly the matrix-degrading enzymes MMP-2 and MMP-9 (Hollestelle *et al*., 2004). TLR4 can act by stimulating macrophages to produce MMPs, contributing to fibrous cap thinning, or leading to smooth muscle cell (SMC) apoptosis; this reduces ECM production and subsequently weakens the fibrous cap and causes plaque rupture. In this context, patients with acute coronary syndromes or coronary arteriosclerosis showed increased TLR4 expression on circulating monocytes, compared with control patients (Methe *et al*., 2005; Geng *et al*., 2006); high expression of TLR4 was also found at the site of ruptured plaques in patients with acute myocardial infarction (Ishikawa *et al*., 2008).

In light of these findings, because atherosclerotic plaque instability is associated with the presence in the plaque of inflammatory cells producing pro-inflammatory cytokines and MMPs, causing matrix turnover, and because TLR4 is associated with both inflammation and matrix breakdown, TLR4 signaling appears to be important in plaque progression and instability, and in the subsequent plaque rupture.

In the context of atherosclerosis, during which inflammatory events are accompanied by oxidative stress, alongside the classic endogenous ligands, oxidized lipids also appear to be capable of activating TLR4 and of modulating an inflammatory response. In this connection, it has been shown that mmLDLs and their active components can trigger cytoskeletal rearrangements dependent on TLR4, which lead to enhanced uptake of lipoproteins by macropinocytosis, in turn leading to intracellular lipid accumulation (Choi *et al*., 2009); alternatively, these lipid compounds can contribute to early-stage intimal foam cell formation, by increasing the expression of scavenger receptors (Doyle *et al*., 2004). In addition, it has been shown *in vivo* that endothelial adhesion and recruitment of macrophages and other leukocytes in response to mmLDLs are impaired in the absence of MyD88 (Michelsen *et al*., 2004). Moreover, mmLDLs induce TLR4-dependent and TLR4-independent cytokine secretion from macrophages (Miller *et al*., 2005); they have also been reported to induce IL-1β, IL-6, and IL-10 secretion in human monocytes and macrophages, in a process involving activation of TLR2 and TLR4 (Chávez-Sánchez *et al*., 2010). Regarding oxLDLs, it has been found that they induce upregulation of TLR4 expression and its signaling pathway, in human monocytes/macrophages *in vitro*, contributing to the TLR4-dependent lipid accumulation and inflammatory process in the arterial wall (Xu *et al*., 2001; Yang *et al*., 2011). In patients with unstable angina, oxLDLs may contribute to monocyte overproduction of some cytokines and above all to that of IL-6 and IL-1β, by upregulating TLR4 expression (Fratta Pasini *et al*., 2007). Moreover, activation of the receptors TLR4 and CD36 by oxLDLs in macrophages induces secretion of various cytokines and foam cell formation (Chávez-Sánchez *et al*., 2014). It has also been shown that oxLDLs regulate the expression of various inflammatory cytokines via the TLR4/NF-κB pathway in arterial SMCs from wild-type mice. This effect was significantly weakened in cells derived from TLR4 knockout mice (Yang *et al*., 2014).

Because mmLDLs and oxLDLs act as endogenous ligands of TLR4, we can speculate that, among the various oxidized lipids carried by mmLDLs or oxLDLs, the principal agents of TLR4 activation, promoting inflammatory molecule release, might be oxysterols, unsaturated aldehydes, and oxidized phospholipids. Nowadays, however, few studies have addressed this point and those that have report contradictory results. For example, in aortic ECs, oxidized 1-palmitoyl-2-arachidonoyl-sn-glycero-3-phosphorylcholine (oxPAPC), one of the main phospholipids of LDL, has been suggested to increase the expression of IL-8, a chemokine important in monocyte recruitment, acting through TLR4 (Walton *et al*., 2003a); contemporarily, the same research group noted that specific oxidation products of oxPAPC may inhibit chemokine induction by TLR4 and TLR2 ligands, in ECs and macrophages (Walton *et al*., 2003b). In primary neuronal cultures, HNE was found to increase TLR4 signaling inducing the degeneration of neurons to amyloid-β and oxidative stress (Tang *et al*., 2008). In contrast, in human macrophage THP-1, the oxysterols 25-OH, 7β-OH, and 7-ketocholesterol may contribute to increasing expression of certain pro-inflammatory cytokines, principally IL-8, but this occurs by mechanisms that are independent of TLR signaling, including TLR4 (Erridge *et al*., 2007). Moreover, in another study, HNE was showed to suppress the activation of signaling pathways and gene expression induced by TLR4 agonist resulting in down-regulation of the phagocytic activity of macrophages (Kim *et al*., 2009). These contradictory results could depend on different cell types, oxidized lipids, and concentrations used.

In light of these findings, the present study aimed to determine the effect on TLR4 activation of 27-OH, reported to be the principal oxysterol responsible for the MMP-9 upregulation (Gargiulo *et al*., 2011) and that of HNE, one of the most reactive unsaturated aldehydes derived from polyunsaturated fatty acid oxidation (Esterbauer *et al*., 1991). These two oxidized lipids are consistently involved in the pathogenesis of atherosclerosis, because they accumulate in the atherosclerotic plaque in considerable amounts and are potent inducers of oxidative stress and inflammatory events (Leonarduzzi *et al*., 2005; Poli *et al*., 2009). Moreover, given the crucial role of inflammatory cytokines and MMPs in plaque rupture, it was also decided to clarify involvement of the TLR4/NF-κB-dependent signaling pathway, activated by 27-OH and HNE, in promoting IL-8, IL-1β, and TNF-α, as well as in MMP-9 production, in human promonocytic U937 cells. Both 27-OH and HNE enhanced expression and protein levels of TLR4 and induced the nuclear translocation of the transcription factor NF-κB, as TLR4 downstream signal (Fig.1). Activation of NF-κB is essential for the regulation of a variety of genes that are involved in the inflammatory responses of cells critical to atherogenesis. The effects exerted by 27-OH and HNE on the TLR4/NF-κB signaling pathway significantly promoted expression and protein levels of IL-8, IL-1β, and TNF-α, that is, cytokines involved in plaque instability, as was clearly demonstrated using an antibody against TLR4 or TLR4 siRNA (Figs3 and 4), or parthenolide, an inhibitor of NF-κB nuclear translocation (Fig5 and 6). Because TLR4 may contribute to matrix breakdown, leading to higher expression levels of MMPs, it was then investigated whether 27-OH and HNE might upregulate MMP-9, which is consistently implicated in the pathophysiology of plaque instability and rupture, via TLR4/NF-κB-dependent pathway. Both compounds markedly induced expression and protein levels of MMP-9, the induction being significantly lessened upon using an anti-TLR4 antibody or TLR4 siRNA, or the inhibitor (parthenolide) of NF-κB (Fig.7).

These observations clearly indicate that 27-OH and HNE are able to promote production of the inflammatory cytokines studied here, and of MMP-9, through the TLR4/NF-κB signaling pathway. The results are in agreement with other studies, for example, *in vitro* studies have shown that TLR2 and TLR4 ligation induces the secretion of MMP-9 in monocytes and mast cells, which are present in the walls of human coronary arteries (Ikeda & Funaba, 2003; Gebbia *et al*., 2004). It has also been demonstrated that activation of TLR4 induces MMP-9 expression in human aortic SMCs through the TLR4/NF-κB signaling pathway (Li *et al*., 2012) and that it has a comparable effect in human umbilical vascular ECs (Paolillo *et al*., 2012).

In this study, TLR4 signaling thus appears to be important in plaque progression and instability, although other signaling pathways may be activated by lipid oxidation products, and they may be interconnected. In a previous study, the present authors showed that an oxysterol mixture, of composition similar to that found in advanced human carotid plaques (Leonarduzzi *et al*., 2007), upregulates MMP-9 through a sequence of events: overproduction of ROS, leading to activation of the MAPK signaling pathways, via protein kinase C, and enhancement of DNA binding of NF-κB, and activator protein-1 (AP-1) (Gargiulo *et al*., 2011). Further, it is known that an inflammatory state can contribute to MMP production. Various inflammatory molecules (e.g., IL-1β, IL-8, IL-12, IL-18, and TNF-α) may operate a control level of MMP production, regulating their expression at the transcriptional levels and their cell release. It has been reported that oxLDLs and oxysterols (mainly 25-hydroxycholesterol) can lead to an imbalance between MMPs and tissue inhibitors of MP (TIMPs), by inhibiting TIMP-1 expression in macrophages. This inhibition is partially mediated by IL-8 (Moreau *et al*., 1999). In this connection, it was observed in this study that the increased inflammatory cytokines (IL-8, IL-1β, and TNF-α) act on MMP-9 by upregulating its expression and synthesis, thus sustaining the release by vascular cells of this matrix-degrading enzyme and contributing to plaque instability (Fig.8). In support of these findings, a recent study reported that activation of the TLR4/NF-κB pathway in microvascular ECs triggered marked upregulation of inflammatory molecules, which play a major role in the cross talk between ECs and monocytes/macrophages, leading to upregulated MMP expression, mainly via IL-6 secretion (Lu *et al*., 2012). Moreover, TNF-α induces expression of MMP-2 and MMP-9 in vascular SMCs, through the NF-κB pathway (Zhong *et al*., 2014).

Taken together, these data support the important role of 27-OH and HNE in atherosclerosis instability, and for the first time, we have demonstrated that these oxidized lipids act as endogenous ligands of TLR4. The compounds 27-OH and HNE contribute to both inflammation and matrix breakdown through activation of TLR4 and its downstream signaling. It thus appears that activation of TLR4 is fundamental for atherosclerosis due to its participation in the production of inflammatory cytokines and MMP-9, although other signaling pathways may also be involved. These data therefore support the hypothesis that atherosclerosis is a consequence of a complex inflammatory process, in which immune response might be involved. Moreover, because TLR4/NF-κB appears to play a predominant role in inflammation and matrix degradation in atherosclerosis, these observations may provide a foundation for the development of innovative therapeutic strategies, such as antagonists of TLR4 or TLR4 siRNAs, for the prevention and therapy of atherosclerosis and its complications.

## Experimental procedures

### Cell culture and treatments

The human promonocytic cell line U937 was cultured in RPMI 1640 medium supplemented with 10% fetal bovine serum (Invitrogen, Life Technologies, Monza, Italy), 100 U mL^-1^ penicillin, 100 μg mL^-1^ streptomycin, and 2 mm glutamine (Sigma-Aldrich, Milan, Italy) at 37 °C with 5% CO_2_. The cells were dispensed at 1 × 10^6^/mL and made quiescent through overnight incubation in serum-free medium. They were then placed in RPMI 1640 medium with 2% fetal bovine serum and treated with 6 μm 27-OH (Avanti Polarlipids, Alabaster, AL, USA) or in serum-free RPMI 1640 medium and treated with 5 μm HNE (Alexis, Vinci-Biochem, Vinci, Italy). In certain experiments, cells were co-treated with anti-TLR4 (0.2 μg mL^-1^), anti-IL-8 (0.04 μg mL^-1^), anti-IL-1β (0.04 μg mL^-1^), or anti-TNF-α (0.04 μg mL^-1^) antibodies (Santa Cruz Biotechnology, Santa Cruz, CA, USA). In others cases, cells were pretreated for 1 h with 1 μm parthenolide (PTN) (Sigma-Aldrich), a specific inhibitor of NF-κB nuclear translocation. Final concentrations and incubation times for all experiments are reported in the figure legends.

### RNA extraction

Total RNA was extracted from cells using TRIzol reagent (Applied Biosystems, Life Technologies), following the manufacturer’s instructions, after treatment for specified times with 27-OH or HNE. RNA was dissolved in RNase-free water with RNase inhibitors (RNase SUPERase-In; Ambion, Life Technologies). The amount and purity (A_260_/A_280_ ratio) of the extracted RNA were assessed spectrophotometrically.

### cDNA preparation and real-time RT–PCR

cDNA was synthesized by reverse transcription from 2 μg RNA with a commercial kit and random primers (High-Capacity cDNA reverse transcription kit; Life Technologies), following the manufacturer’s instructions. Singleplex real-time RT–PCR was performed on 40 ng of cDNA using TaqMan gene expression assay kits prepared for human TLR4, IL-8, IL-1β, TNF-α, MMP-9, and β-actin and TaqMan Fast Universal PCR master mix, and analyzed by a 7500 Fast real-time PCR system (Applied Biosystems, Life Technologies). The oligonucleotide sequences are not revealed by the manufacturer because of proprietary interests. The cycling parameters were as follows: 20 s at 95 °C for AmpErase UNG activation, 3 s at 95 °C for AmpliTaq Gold DNA polymerase activation, and 40 cycles of 3 s each at 95 °C (melting) and 30 s at 60 °C (annealing/extension). The fractional cycle number at which fluorescence passes the threshold in the amplification plot of fluorescence signal vs. cycle number was determined for each gene considered. The results were then normalized to the expression of β-actin, as housekeeping gene. Target gene expression was quantified relatively with a mathematical method proposed by Livak & Schmittgen (2001).

### siRNA transfection

Small interfering RNA (siRNA) was used for transient gene knockdown studies. The siRNAs used were TLR4 s14195 and siRNA #1 for the negative control (scramble siRNA) (Applied Biosystems, Life Technologies). Negative control corresponds to a siRNA with nonspecific sequence. Transfection of TLR4-specific and control siRNAs was performed following the manufacturer’s instructions. Briefly, 50 nm of siRNAs was mixed with 25 μL of transfection reagent solution (NeoFX, Applied Biosystems, Life Technologies) and left at room temperature for 10 min in RPMI medium with 1% fetal bovine serum and without antibiotics. After 24 h of reverse transfection, the cells (4 × 10^4^ 500 μL^−1^) were incubated with 27-OH or HNE for 6 or 24 h. For gene expression analysis, total RNA was isolated from the cells and used for quantitative RT–PCR as described above. The transfection efficiency, validated by quantitative RT–PCR, was approximately 60%.

### Western blotting

Whole-cell extracts were prepared in ice-cold lysing buffer (50 mm Tris–HCl, pH 7.4, 50 mm NaCl, NP-40 1%, Triton X-100 1%, 0.5 mm EDTA, sodium deoxycholate 1%) containing protease inhibitors (5 mm DTT, PMSF 0.1%, aprotinin 1%). Total proteins (65 μg) were separated by electrophoresis in 8% denaturing SDS/polyacrylamide gel and then transferred to Hybond ECL nitrocellulose membrane (GE Healthcare Europe, Milan, Italy). After saturation of nonspecific binding sites with 5% nonfat milk in Tris-buffered saline (TBS) 1x-Tween 20 0.05%, the membrane was immunoblotted overnight at 4 °C with the primary antibody against TLR4 (1:500) and subsequently probed with an anti-goat secondary antibody (1:1000) (Santa Cruz Biotechnology Inc.,) overnight at 4 °C. The membrane was stripped (Restore Western Blot Stripping buffer, Pierce Biotechnology, Rockford, IL, USA) and re-immunoblotted with anti-actin primary antibody (1:7500) and then with anti-rabbit secondary antibodies (1:5000) (Santa Cruz Biotechnology Inc.). The immunoreactive bands were visualized through enhanced chemiluminescence using the ECL-plus kit (GE Healthcare Europe) following the manufacturer’s protocol.

### Analysis of TLR4, p65, IL-8, IL-1β, and TNF-α by immunofluorescence and detection by confocal laser microscopy

After treatments, cells were transferred onto glass slides (8 × 10^4^ cells/slide) by cytocentrifugation. Specimens were fixed in cold methanol for 10 min and permeabilized with 0.1 m PBS 0.4% Triton X-100 solution. Cells were then incubated with a 100 mm sodium cyanoborohydride-reducing agent for 10 min at 37 °C. After blocking nonspecific sites of binding with 0.1 m PBS containing 5% goat serum, 3% BSA, and 0.3% Tween 20, for 1 h at room temperature, slides were incubated in the presence of primary antibodies against TLR4 (1:50), p65 (1:50), IL-8 (1:100), IL-1β (1:50), and TNF-α (1:50) (Santa Cruz Biotechnology) and then with specific secondary antibodies (1:300) conjugated with fluorescein isothiocyanate (FITC) or tetramethylrhodamine isothiocyanate (TRITC) fluorochromes (Alexa Fluor, Molecular Probes, Life Technologies). Slides mounted with glycerol and distilled water (1:1) were observed through an LSM 510 confocal laser microscope (Carl Zeiss SpA, Arese, Milan, Italy) equipped with an inverted microscope with Plan-NEOFLUAR lenses (40x/0.75).

### Evaluation of protein levels by Bio-Plex® system or ELISA

After treatments, cells were lysed and cytosolic proteins were stored for protein levels assessment. Protein levels of IL-8, IL-1β, and TNF-α were measured using a magnetic bead-based assay designed for the simultaneous detection of the cytokines, reading in a Bio-Plex® system dual laser instrument (Bio-Rad). Assays were performed following the manufacturer’s instructions using 50 μg protein lysate per well.

Protein levels of IL-8, IL-1β, TNF-α, and MMP-9 were also quantified using: Human IL-8 ELISA MAX™ Standard Set (BioLegend, San Diego, CA, USA) for IL-8, Human IL-1 beta ELISA Ready-SET-Go!® (eBioscience, San Diego, CA, USA) for IL-1β, Human TNF-α Instant ELISA (Bender MedSystems, Wien, Austria) for TNF-α, and the DuoSet ELISA kit (R&D System, MN, USA) for MMP-9, following the manufacturer’s instructions. The plates were read at 450 nm with wavelength correction of 550 nm in a microplate reader (Model 680 Microplate Reader, Bio-Rad, Hercules, CA, USA).

The protein concentrations were extrapolated from the standard curve.

### Statistical analysis

All values are expressed as means ± standard deviation (SD). Statistical analysis of the data was by one-way anova with Bonferroni’s post-test for multiple comparisons. Differences with *P* < 0.05 were considered statistically significant. Statistical calculations were made with the GraphPad InStat3 software package (GraphPad Software, San Diego, CA, USA).
